# Lightweight Advanced Deep Neural Network (DNN) Model for Early-Stage Lung Cancer Detection

**DOI:** 10.3390/diagnostics14212356

**Published:** 2024-10-22

**Authors:** Isha Bhatia, Aarti, Syed Immamul Ansarullah, Farhan Amin, Amerah Alabrah

**Affiliations:** 1 Department of Computer Science and Engineering, Lovely Professional University, Phagwara 144411, Punjab, India; ishabhatia91@gmail.com (I.B.); aarti.1208@gmail.com (A.); 2 Department of Management Studies, University of Kashmir, North Campus, Delina, Baramulla 193103, Jammu & Kashmir, India; syedansr@gmail.com; 3 School of Computer Science and Engineering, Yeungnam University, Gyeongsan 38541, Republic of Korea; 4 Department of Information Systems, College of Computer and Information Science, King Saud University, Riyadh 11543, Saudi Arabia

**Keywords:** deep learning, lung carcinoma, CT image, lung cancer, convolutional neural networks, image classification

## Abstract

**Background:** Lung cancer, also known as lung carcinoma, has a high mortality rate; however, an early prediction helps to reduce the risk. In the current literature, various approaches have been developed for the prediction of lung carcinoma (at an early stage), but these still have various issues, such as low accuracy, high noise, low contrast, poor recognition rates, and a high false-positive rate, etc. Thus, in this research effort, we have proposed an advanced algorithm and combined two different types of deep neural networks to make it easier to spot lung melanoma in the early phases. **Methods:** We have used WDSI (weakly supervised dense instance-level lung segmentation) for laborious pixel-level annotations. In addition, we suggested an SS-CL (deep continuous learning-based deep neural network) that can be applied to the labeled and unlabeled data to improve efficiency. This work intends to evaluate potential lightweight, low-memory deep neural net (DNN) designs for image processing. **Results:** Our experimental results show that, by combining WDSI and LSO segmentation, we can achieve super-sensitive, specific, and accurate early detection of lung cancer. For experiments, we used the lung nodule (LUNA16) dataset, which consists of the patients’ 3D CT scan images. We confirmed that our proposed model is lightweight because it uses less memory. We have compared them with state-of-the-art models named PSNR and SSIM. The efficiency is 32.8% and 0.97, respectively. The proposed lightweight deep neural network (DNN) model archives a high accuracy of 98.2% and also removes noise more effectively. **Conclusions:** Our proposed approach has a lot of potential to help medical image analysis to help improve the accuracy of test results, and it may also prove helpful in saving patients’ lives.

## 1. Introduction

Predicted figures for 2022 in the U.S. include 609 new tumor cases and fatalities, 360 cancer deaths, and 1.9 million new cancer cases [1,2,3]. The causes of lung cancer are smoking cigarettes [1,2,3,4], one’s living situation, one’s diet, one’s underlying chronic illnesses, and genetics. The primary cause of lung cancer [5] is a common malignant tumor [6]. It is an abnormality in the bronchial mucosa. As a result, lung cancer manifests [7]. The patients typically experience discomfort in their throats and lungs [8], including coughing, dysphonia, chest pain, and other sorts of warning signs [9]. These symptoms might also be accompanied by additional issues such as lung discomfort and malignant pleural effusion. As their lung cancer worsens, patients may have a variety of symptoms. Chest pain, voice changes, and a persistent cough are common symptoms. Furthermore, issues like localized discomfort and malignant pleural effusion—a condition in which fluid builds up in the pleural area surrounding the lungs—may arise. The impact of the cancer on the lung tissues and pleura causes these problems, underscoring the significance of early discovery for efficient management and therapy. Certain types of primary lung cancer, such as large-cell carcinoma, are included in the more general classification “Primary lung cancer or malignant”. All types of primary lung cancer, such as adenocarcinoma, squamous cell carcinoma, small-cell lung carcinoma, and large-cell lung carcinoma, fall under this category. Compared to other forms of lung cancer, large-cell carcinoma has significantly larger cells. Although it can occur anywhere in the lung, LCLC is most frequently detected on the outermost part of the lung. Compared to other NSCLC kinds, it tends to develop and spread faster; therefore, early detection and treatment are essential. In this situation, early lung cancer [10] detection is essential [11] for saving an individual’s life [3]. A common clinical symptom is pulmonary nodules, which are often spherical or irregular lesions in the lung with a diameter of ≤3 cm [12]. They can be solitary or numerous, with boundaries that are obvious or obscure. Currently, the majority of pulmonary nodule evaluation techniques rely on traditional imaging [13]. In computed tomography (CT) examinations, the results need to be processed, and accurate lung cancer is found using mathematical tools from artificial intelligence [9,14,15,16]. In addition, it is evident from the literature that deep reinforcement learning [17] and adaptive adversarial networks are used [18]. The process of evaluating medical images to diagnose cancer disease includes a post-processing phase that involves capturing suitable characteristics after applying the preprocessing and image segmentation stages. In the literature, various methods have been proposed, including feature-grabbing. The most popular techniques are region-merging, opening and closing operations, island removal, border extension, and smoothing. Using deep learning techniques, the phases classify medical images into cancer groups [19] according to the properties and were extracted during the post-processing stage. Diverse principles have guided the development of various deep learning procedures and can be broadly categorized into four groups. Using the features retrieved from the photos, the deep learning classifier is trained and tested to identify different forms of cancer during the classification phase [20]. Using photos from the training dataset, a deep learning classifier is trained in the first stage. Based on the attributes that were retrieved, the accomplished deep learning model uses the required number of training iterations to predict the type of cancer in unknown photos. Recurrent neural networks (RNNs), long short-term memories (LSTMs) [21], convolution neural networks (CNNs), and gated recurrent units (GRUs) are the most broadly utilized deep learning methods for melanoma uncovering [22,23]. RNN training is difficult because of the gradient problem. The issue of vanishing gradients affects RNNs. The RNN uses the gradients to carry information, and when the gradient diminishes too much, the parameter updates lose their significance. Long-data-sequence training becomes challenging as a result [23]. LSTMs need larger amounts of training data to function well. Secondly, they are not appropriate for online learning tasks when the input data are not a sequence, like prediction or classification tasks. Third, when training on massive databases, LSTMs may be slow [24]. Despite their strengths, CNNs are limited by their lengthy training periods, large dataset needs, low inference times, dynamic environments, and hardware dependence. GRUs, the model’s complexity makes the training process both time and time-consuming, not more focused on accuracy [21]. The state-of-the-art methods have low accuracy and large data sizes. In addition, most of the techniques were not effective, particularly in identifying cancer in its early stages. Most of them frequently rely on sizable labeled datasets, which are expensive and time-consuming [23]. We found that segmentation and classification techniques [19,25] were used to analyze and predict lung cancer at its early stages, but they did not focus on accuracy and lightweight size. Therefore, to overcome these challenges [26], herein we propose a lightweight deep neural network (DNN) model for early-stage lung cancer detection [5,6,7,8]. Several strategies for lung cancer early identification and prediction have been investigated in earlier studies. These approaches involve sophisticated deep learning models, mathematical tools from artificial intelligence, and conventional imaging techniques. Many obstacles remain in the face of these efforts, including the high false-positive rates, low precision, and expensive computational resource requirements.

Deep learning methods, such as Convolutional Neural Networks (CNNs), Long Short-Term Memory (LSTM) networks, and other models, have been used in recent advances. Although these techniques have potential, they frequently have issues with accuracy, processing speed, and the capacity to manage big datasets effectively. Our study’s goal is to overcome these constraints by putting forth a novel strategy that integrates the deep continuous learning-based deep neural network (SS-CL) and weakly supervised dense instance-level lung segmentation (WDSI) deep neural network models. Our objective is to create a low-weight, high-precision model that detects lung cancer in its early stages and outperforms current approaches in terms of accuracy, efficiency, and low computing load.

Our goal is to reduce computing demands and increase the accuracy of lung cancer detection by the integration of different methodologies, which is essential for real-world applications in medical imaging and patient care.

### Key Contributions of Our Study

The explanation details and contributions of our proposed model are given below.

- In this research, we propose a lightweight model to overcome noisy regions, such as graininess, tissues [27], and vessels, namely a Ricker Wavelet Iterative Center Weighted Median Filter (RWICWM).
- To reduce false positives in the disease prediction accuracy, Sørensen–Dice Index-based K-means clustering has been suggested.
- To detect varying size nodules in lungs, Light Spectrum Optimizer-based pulmonary nodule detection (WDSI-LSO) has been used.
- To differentiate lung parenchyma from the segmented lung, a sliding window strategy has been suggested.
- To screen patients for future analysis, a risk screening has been made based on solitary nodule detection using PLCOm2012.
- To appropriately classify lung cancer with high accuracy, a semi-supervised and contrastive learning [28]-based Deep Neural Network (SSCL-DNN) has been proposed.
- The proposed algorithm evolved using a hybrid method and was compared to other algorithms, such as MLP, CNNs, and RNNs. Google Deep Mind was the first to use reinforcement learning technology in 2013.

Our research presents a novel strategy to improve lung cancer risk assessment and diagnosis by combining many cutting-edge approaches. A semi-supervised Contrastive Learning-based Deep Neural Network (SSCL-DNN) for robust classification, a novel combination of Sørensen–Dice Index-based K-means clustering and a Weibull Distributed Scale factor integrated-Light Spectrum Optimizer (WDSI-LSO) for improved nodule detection, and the Ricker Wavelet Iterative Center Weighted Median Filter (RWICWM) for superior noise reduction are some of the key features. Our work addresses several important issues: it provides a revised classification system, improves risk prediction with PLCOm2012 integration, increases accuracy in detecting nodules of different sizes, and tackles noise-handling concerns. In particular, our strategy closes important gaps in existing approaches, like poor noise control, low detection accuracy, poor risk prediction, and inefficient classification. The format of the paper is as follows: a summary of previous approaches is given in Section 2, the proposed model is discussed in Section 3, the outcome and discussion of the suggested methodology are discussed in Section 4, and the conclusion is discussed in Section 5.

## 2. Literature Survey

Sun et al. [1] combined the use of Wavelet feature descriptors with an artificial neural network. The wavelet transform produces the computed statistical properties, which are then used as input parameters for the neural network classifier. These properties include autocorrelation, entropy, contrast, and energy [24]. However, because this method uses artificial neural networks (ANNs) for classification, which have a slow learning curve, computing time is important. The Lung Image Database Consortium image collection (LIDC-IDRI) [28] in The Cancer Imaging Archive (TCIA) database was evaluated by Tajidini et al. [2] using the SVM-LASSO model [29]. PNs’ malignancy could be predicted with the two CT radiomic features—the directional variation in the local area. Nonetheless, there were no radiomic characteristics that distinguished spiculated or lobulated borders in particular. Sankar and George invented the idea of Regression Neural Network (RNN) segmentation [3,30]. It offered a high degree of identification of nearby lesions [31] of comparable intensity. Additionally, due to juxta vascular and juxta pleural nodes, boundary recognition [32] is precise through segmentation. An RNN (Regression Neural Network) is a learning procedure that has made it possible to bypass the difficulties involved in programmed lesion finding. However, the main focus of the reported RNN approach is the precision of lung parenchyma segmentation and the precise detection [4] of boundaries for juxtavascular and juxtapleural diseases. The lung segmentation approach was presented by Khodadoust et al. [4] in two ways: First, a lung segmentation method based on morphology and thresholding was introduced [27]. The other contribution is the way [33] the nodule-type identification problem is presented [31], along with a basic method of doing it. This study has presented the problem and solution approach in the context of the lung segmentation method [34] because there is currently no ground-truth information available regarding nodule types. However, since the classification divides the nodules as fat, it is incorrect to include the nodules when they are linked to the pleural surface or vascular area. An advanced feature driven approach is presented by Wang et al. in [5]. In this research study, they presented a research on mining of CT Images. They conducted an analysis in this research [5]. As the study object, pulmonary nodule CT scans were gathered, and the image characteristics were extracted using a feature extraction model for the lung nodule, which is based on EM, i.e., expectation maximization [20,35]. However, it is challenging to identify them in the early stages [5] because the aforementioned symptoms lack classical specificity [12] and the majority of patients often do not exhibit clear melanoma uncovering at that time [36]. For premature-stage lung adenocarcinomas [29], this study developed a two-stage risk categorization system [37]. The results demonstrated the following: (1) senior radiotherapists with more than fifteen years of experience were outperformed by the DNN; (2) the relationship between the radiologist’s experience and the diagnostic performance showed a positive trend [27]; and (3) the DNN’s performance was negatively impacted by low CT image resolution [1,9]. The deep learning technique was demonstrated to be an effective means of categorizing GGN risks [18], potentially enhancing future methods for GGN diagnosis [38] and simplifying assisted- or second-read procedures [29,39,40]. Three different kinds of deep neural networks were constructed by Song et al. [34] to classify lung cancer. With a few modifications, these networks were used to categorize benign and malignant lung with 84.32% specificity, 84.15% accuracy, and 83.96% sensitivity [41]. To diagnose lung cancer from CT data, Isha et al. [32] provided a deep residual learning [30] strategy that extracted attributes using UNet and ResNet models [26,42]. Several classifiers, including Random Forest and XGBoost, were used for the feature set, and by assembling the individual predictions [11], an accuracy of 84% was obtained. An overview of the computer-aided detection (CAD) methods for lung cancer in computed tomography nodules in CT scans is provided. The displayed CNN network was given by Deb et al. Many research teams have recently investigated whether lung [43] disorders can be diagnosed with MIT [44,45]. However, there are still a lot of obstacles to overcome before MIT technology can be widely employed as a commercial imaging tool [28]. These obstacles include poor image quality, the high expense of computational electromagnetic models, and a dearth of measurement techniques. A hybrid-based deep learning system for the categorization of Otitis Media with Effusion (OME) based on eardrumotoendoscopic pictures [46]. The suggested model extracted and selected characteristics by combining the Gaussian method [34,47] with Neighborhood Component Analysis (NCA). The testing [47] findings demonstrated that the proposed model attained a high accuracy of 94.8% based on 910 image datasets. Harun Bingol presented a novel technique for detecting cervical cancer in Gauss-enhanced pap smear images [43] by employing a hybrid CNN model. When the suggested model’s performance was evaluated using a dataset of 1000 photos, it outperformed several other current techniques with an accuracy of 93.6% [29]. We will work on an automatic system to ascertain the patient’s condition—whether they are healthy or not—and to detect diseases early (in other words, to identify the cancerous node at the very beginning).

## 3. Proposed Methodology

**Figure 1:**
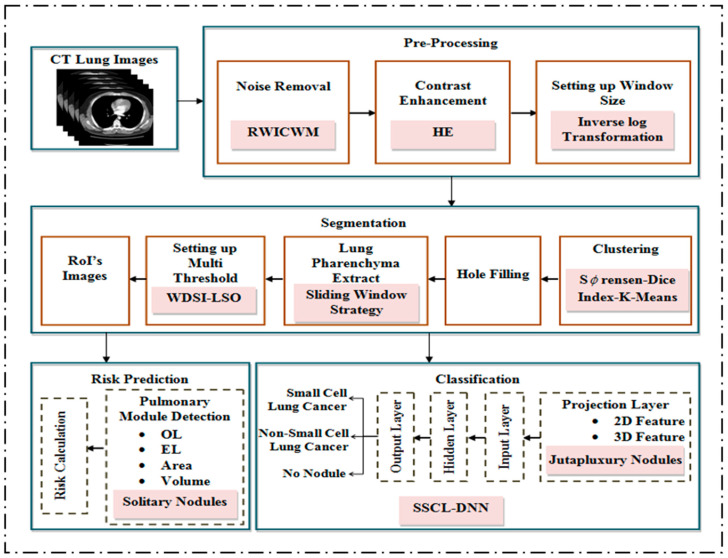
Block schematic of the proposed model.

This proposed framework is shown in Figure 1 and works under the following phases: 1. Preprocessing (Noise elimination and Contrast enhancement). 2. Segmentation phase. 3. Risk score prediction phase. 4. Classification phase.

### 3.1. Processing Phase

First, input lung CT (computed tomography) scan images are collected from the LIDC (Lung Image Database Consortium) dataset in the DICOM (Digital Imaging and Communications in Medicine) format and are given to the Ricker Wavelet Iterative Center Weighted Median Filter (RWICWM). The suggested filter improves the variance field estimation by making use of the image ricker wavelet coefficients’ inner- and inter-scale dependence. By smoothing the noisy wavelet coefficient variances iteratively, this filter maintains the edge information found in the large-magnitude wavelet coefficients [28]. Here, the results will be evaluated for parameters such as PSNR (peak signal-to-noise ratio), MSE (Mean Square Error), and SSIM (Structured Similarity Index Method) with conventional de-noising filters, such as a Gaussian filter, guided filter, and wiener filter. A histogram equalization technique is adopted for contrast enhancement [4,48]. After determining the proper window size level, the slope and intercept are rescaled using the inverse log transformation [32].

(1) $$\;\;I_{RWICWM=RWICWM\left(I_{orignal}\right)}$$

where O_riginal_ is the original image and I_RWICWM_ is the filtered image.

Filter-based image de-noising: The following is a mathematical representation of the de-noising procedure with a filter F:

(2) $$I_{denoised}=F\left(I_{noisy}\right)$$

where *I_de-noised_* is the de-noised image. *I_noisy_* is the noisy input image (Table 1).

Equalization of the histogram: The contrast of the de-noised image is improved through the use of histogram equalization. The operation can be modeled as follows [49]:

(3) $$I_{equalized\;=\;HE\left(I_{denoized}\right)\;}$$

where I equalized is the histogram-equalized image. HE (⋅) denotes the histogram equalization operation.

Quality Measures: To evaluate how well the de-noising and equalization procedure performs, the following quality measures can be computed:

- Peak Signal-to-Noise Ratio (PSNR)

(4) $$PSNR=10.log_{10}\left(\frac{MAX}{MSE}\right)$$

where MAXMAX is the highest possible pixel value (for example, 255 in 8-bit pictures). MSEMSE is the Mean Squared Error between the original image *I* and the equalized image *I _equalized_*:

(5) $$\mathrm{MSE}=1.\sum =1\sum \left(()-,\right)2\mathrm{MSE}=M.N1\sum i=1M\sum j=1N\left(I\mathrm{original}\left(I,J\right)-I\mathrm{equalized}\left(i,j\right)\right)2$$

### 3.2. Segmentation Phase

Step 1: The preprocessed images will be computed for various intensities and then given to Sørensen–Dice Index K-means clustering; after that, the cluster centers will be initialized, and this step is repeated until convergence is reached. Since Euclidean and other distances are not scaled invariants, meaning that the distances computed could be skewed depending on the features’ units, the Sørensen–Dice Index distance is thus employed as the data index distance calculation [6,23]. Sørensen’s initial formula was intended for use with discrete data. It is defined with 2 specified sets, X and Y, as

(6) $$DSE=\frac{2\left|X\cap Y\right|}{\left|X\right|+\left|Y\right|}$$

where the 2 sets of cardinalities, or the number of essentials in every set, are represented by the variables |X| and |Y|. Divide the whole number of elements in each set by the total sum of items that both sets share twice to obtain the Sørensen index. By using the definitions of false negative (FN), true positive (TP), and false positive (FP), one can write boolean data as follows. The cardinalities, or the number of members in each set, of the two sets are represented by the variables |X| and |Y| [25]. Divide the overalls um of elements in each set by the total sum of items that both sets share twice to obtain the Sørensen index. By using the definitions of true positive (TP), false positive (FP), and false negative (FN), one can characterize boolean data as follows [11,33]:

(7) $$DSE=\frac{2TP}{2TP+FP+FN}$$

Conversely, true positives are only counted once in the numerator and denominator of the Jaccard index. The similarity quotient, or DSC, has a range of 0 to 1 [9,50]. It can be seen as a set-level similarity metric. The established operations can be described in standings of vector operations over binary vectors a and b, which is similar to the Jaccard index:

(8) $$Sv=\frac{2\left|a\cdot b\right|}{\left|a\right|+\left|b\right|}$$

This provides a broader similarity metric across vectors and yields the same result for binary vectors. The coefficient is well defined as two-fold the collective information (intersection) over the total cardinalities for sets X and Y of keywords utilized in information retrieval. Therefore, from each cluster center, centroid intensity will be determined using the Sørensen–Dice Index as a distance measure. For every data point, this process is repeated [45,51]. Conventional clustering techniques, such as the K-means clustering algorithm, centroid-based clustering, and density-based clustering techniques, will be compared with the evaluation results of this clustering method for parameters such as true positive and false positive rates with previous methods.

(9) $$C=K_{means}\left(I_{RWICWM}\right)$$

where the clustered regions are denoted by the C.

Sørensen–Dice Index with K-means Clustering: Using the Sørensen–Dice Index as the similarity metric, we employ K-means clustering to separate the preprocessed image into K clusters. After choosing the K cluster centers, each pixel is subsequently given to the nearest cluster based on the dice similarity.

(10) $$I_{segmented\;}=\left(I\mathrm{preprocessed},\;K,\;Dice\;Similarity\right)$$

The segmented image is represented by the keyword segmented. K-means stands for the K-means clustering procedure. K represents the number of clusters, which normally stand in the foreground and background. The similarity metric used in K-means clustering is dice similarity [52]. The segmented pulmonary nodules are included in the final product, where each pixel is assigned to a cluster depending on how similar it is to the cluster centers using the Sørensen–Dice Index.

Step 2: The result obtained at this stage is then given for pulmonary nodule detection. For pulmonary nodule detection, lung parenchyma is extracted [42,53] using a sliding window strategy, and, from this, lung nodule detection will be carried out using Weibull Distributed Scale factor integrated-Light Spectrum Optimizer-based pulmonary nodule detection (WDSI-LSO) [5,54]. Here, a Light Spectrum Optimizer is taken and, due to its being restricted by the transmission coverage, the scale factor distribution is modified using Weibull distribution. Here, the images’ gray-level values will be initialized, and the goal function is to find the best threshold by histogram analysis and through evaluating each gray level to see which one maximizes the likelihood that the threshold value will occur for each class of probability [24]. Many ROIs with different intensities are obtained from this ideal threshold due to optimal multilayer thresholds. Eventually, these ROIs are concealed using a segmented lung mask to create the collective form of an ROI picture. From this ROI, the features, such as the range of area, volume range, tolerance in overlap (OL) feature, and elongation (EL) feature, are calculated [7,55]. This result will be evaluated for nodule count, Dice Similarity Coefficient (DSC) sensitivity, positive predictive value (PPV), and specificity for U-DNet, NoduleNet, and Faster R-CNN.

(11) $$I_{WDSI-LSO}=WDSI\_LSO\left(I_{RWICWM},\;C\right)$$

where *I_WDSI−LSO_* represents the enhanced image after applying WDSI-LSO. The segmentation process can be visualized as a binary image where pixels corresponding to pulmonary nodules are assigned the value 1 while other pixels are assigned the value 0. A frequent strategy is to employ a thresholding technique, which may be mathematically expressed as 1 if *I_preprocessed_*(x, y) and 0 if *_I_* *_segmented_* (x, y), where *_I_* *_segmented_* (x, y) is the pixel value at coordinates(x, y) in the segmented image. *I_preprocessed_*(x, y) is the corresponding pixel value in the preprocessed image. The threshold is the chosen threshold value [17].

### 3.3. Risk Score Screening

Here, risk screening will be conducted based on solid nodules found at the segmentation result. This will be conducted based on environmental factors such as smoking, family history, and other chronic diseases. Along with this, if positive solid nodules were found, the risk assessment would be performed using PLCOm2012. PLCOm2012 risk assessment also considers the environmental features stated earlier in regard to resulting in high-level and low-level risks. This was carried out to predict the survival rate of the entire database and take precautious care in the future.

(12) $$RiskPLCO_{m}2012=PLCO_{m}2012\left(I_{patient\_data}\right)$$

where *I_patient_data_* contains patient-specific information.

### 3.4. Classification

**Figure 2:**
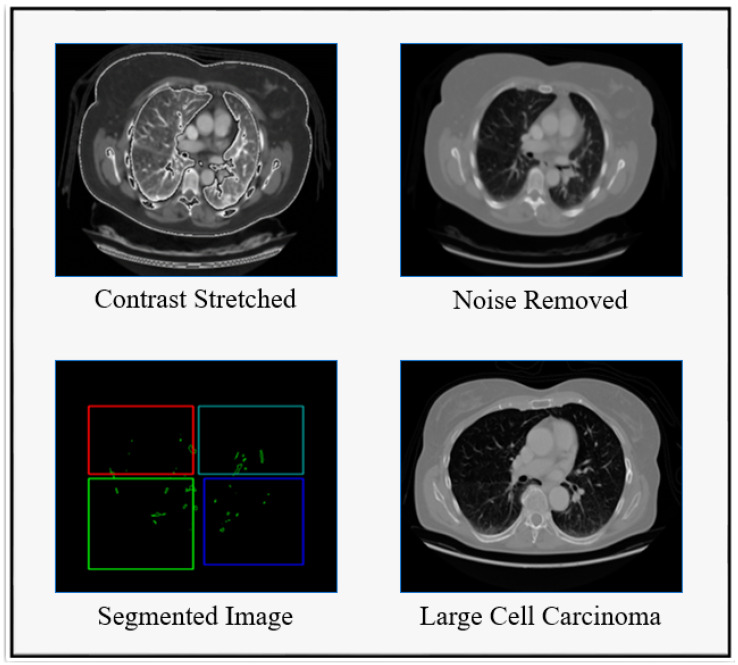
Dataset utilization for cancer detection.

Herein, the preprocessing steps typically include Contrast Stretched, Convex Hull, and Edge Enhanced for resizing the images, normalizing the pixel values and possibly augmenting the data to increase the variety and robustness of the training set, shown below in Figure 2. In the context of lung cancer, labels would indicate the presence or absence of nodules and, if available, the malignancy of detected nodules. In addition, the common architectures used for medical image classification include Convolutional Neural Networks (CNNs), i.e., ResNet, VGG etc. In classification phase, we divide the dataset into training, validation, and test sets. A common split is 70-20-10. Train your DNN using the training set. Use the validation set to tune hyperparameters and avoid overfitting. Afterwards, classification optimizers, like Adam or RMSprop, and risk analyzers are frequently used.

The LUNA16 (Lung Nodule Analysis) CT imaging dataset, which was split into three sets (Test, Training, and Valid), served as the data sample for this study. The classification scheme for lung nodules has been used in the LIDC-IDRI (Lung Image Database Consortium and Image Database Resource Initiative) dataset, which is also referred to as LUNA-16. Nodules are categorized under the system into four groups [24]:

Unknown (No Label): this class includes nodules that do not have an official categorization or label.

Benign, normal, or non-cancerous: these types of nodules are classified as having a low chance of being malignant, meaning they are either benign, normal, or non-cancerous.

Primary lung cancer or malignant lesions: nodules classified as primary lung cancer or malignant lesions fall into this category, suggesting a strong probability that the cancerous growth originated in the lung.

Metastatic Lesion: nodules classified as metastatic lesions fall under this category if the original malignancy did not start in the lungs but rather in another area of the body.

**Figure 3:**
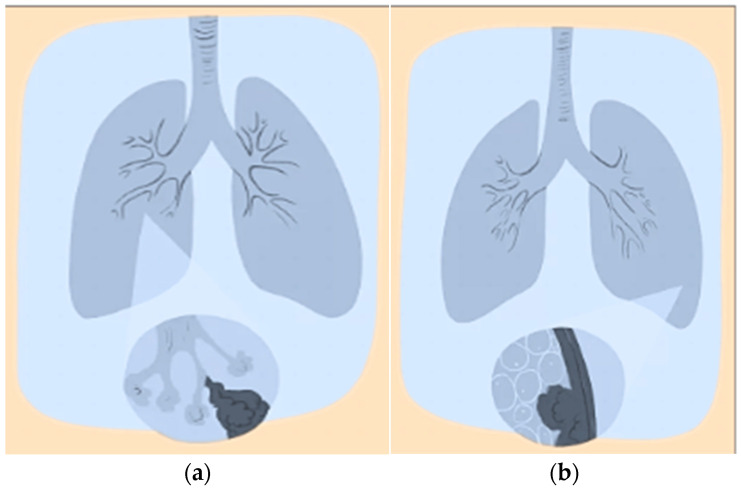
Dataset Utilization for Cancerous Image. (**a**) Large-Cell Carcinoma; (**b**) Squamous Cell Carcinoma.

A single label—normal, large-cell carcinoma, or squamous cell carcinoma—is assigned to each image in the dataset. Throughout the patients’ clinical care schedule, CT scans were performed. Before deep learning models were trained on the dataset, two board-certified physicians rated the diagnosis on the images. Additionally, to ensure that the grading work was performed accurately, a third expert assessed the dataset images. In both the training and test sets, there was a very unequal distribution of classes. In contrast to the training set, certain samples were included in the test set. To create the new training set, the training and test sets were combined, jumbled, and randomly ordered. Figure 3 displays images for cases of squamous cell carcinoma or normal or large-cell carcinoma.

For classification, the segmented nodules were taken as input to the semi-supervised and contrastive learning-based DNN (SSCL-DNN).

*Output SSCL* − *DNN* = *SSCL* − *DNN* (*I_WDSI−LSO_*, *C*, *Risk PLCO_m_*2012)

where Output *SSCL−DNN* represents the output of the neural network, which could include nodule detection scores and risk assessments. By incorporating several projector layers, a contrastive loss term, semi-supervised label propagation, and contrastive learning [1] for classification, a DNN is divided into two sub-networks. 1. The classifier will receive segmented results as a training set. 2. The network has pre-trained models (semi-supervised learning), and it propagates the incoming images into classes, such as small-cell lung melanoma, non-small-cell lung malignancy [56], and no nodule. 3. Then, contrastive learning [28,57] is introduced in the network by adding a projection layer and considering contrast loss. 4. Due to contrasting learning, the input images will be both strongly and weakly augmented, then combined in the projection layer for extraction of 2D features and 3D features. These features will be mapped as feature vectors in the same layer. 5. These results will be given to DNN classification layers, and the upshot will be non-small-cell lung melanoma [7], small-cell lung malignancy, as well as no nodule [58] (Table 2).

The performance will be evaluated for error rate, loss, and accuracy with ResNet, Inception-v3, and semi-supervised deep learning.

Pulmonary Nodule Detection Score = SSCL-DNN (RWICWM + K-means (WDSI-LSO) + PLCOm2012 risk assessment) (Algorithm 1).
**Algorithm 1:** Proposed Algorithm**Input:** selected features $\left(Fea_{sele}\right)$ **Output:** output categorized as either abnormal or normal **Begin** **Initialize** selected features $\left(Fea_{sele}\right)$, weight w **For** all training steps **do** **Compute** Logistic function **Perform** convolution layer $con_{lyr}=\mu \sum Fea_{sele}•w$ **Perform** max pooling layer **Process** fully connected layer $full_{lyr}=\mu \left(pol_{lyr},w+pol_{lyr}\right)$ **End For** **Return** classified output as Normal or Abnormal **End**

Lastly, the output is categorized as normal and abnormal by logistics. An additional risk screening procedure was used to determine whether the classified output was anomalous [22,52].

### 3.5. Dataset Description

The Lung Image Database Consortium image collection serves as the dataset for this proposed work (LUNA-16) [28]. Thoracic computed tomography (CT) scans with marked-up, labeled lesions are used for both lung cancer screening and diagnosis [6]. This 1018-case data collection was produced in collaboration with seven university organizations and eight medical imaging companies [38]. Images from a clinical thoracic CT scan and an XML file containing the annotation process findings, completed in two steps by four seasoned thoracic radiologists, are included for each topic. At the first blinded-read stage, each radiologist independently reviewed each CT image [2] and classified lesions into three categories: “non-nodule > or =3 mm”, “nodule < 3 mm”, and “nodule > or =3 Mm”.

**Figure 4:**
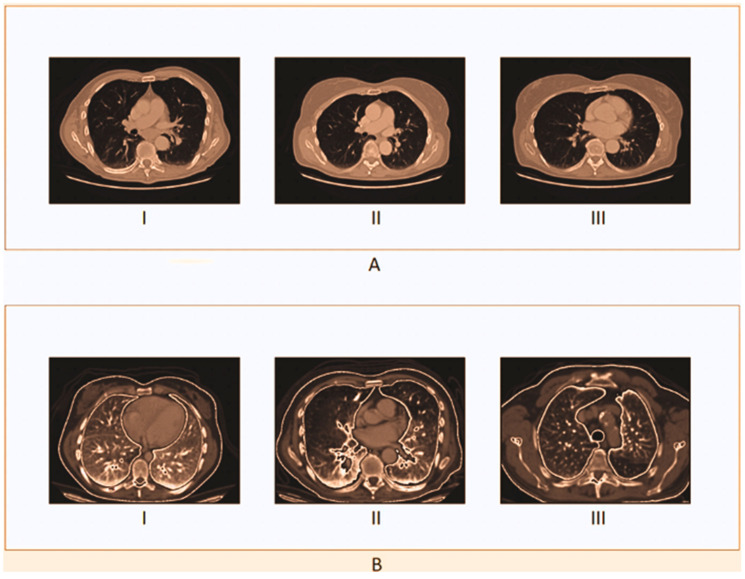

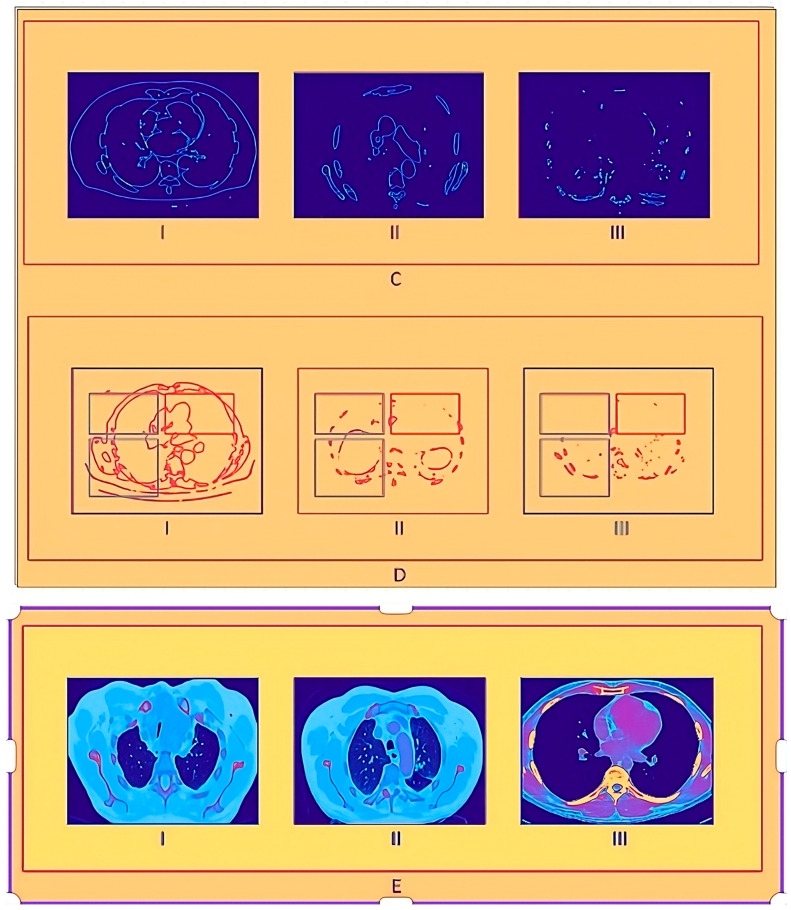
Sample images in the proposed model. (**A**,**B**) The top and bottom images show the original CT sample images and the contrast-stretched images. (**C**,**D**) The top and bottom images show the edge enhancement image and segmentation. (**E**) The images show the classified output (large-cell cancer, squamous cell cancer, and normal).

Here, the sample image of the suggested methodology is shown in Figure 4. In Figure 4A, the original CT sample images are displayed. As can be seen in Figure 4B, the contrast-stretch applied to the input images is preprocessed to remove noise using RWICWM. Next, in the picture of Figure 4C, we see the edge enhancement image. After, in Figure 4D, we see segmentation. Finally, Figure 4E the classified output is large-cell cancer, squamous cell cancer [51], and normal (without carcinoma).

**Figure 5:**
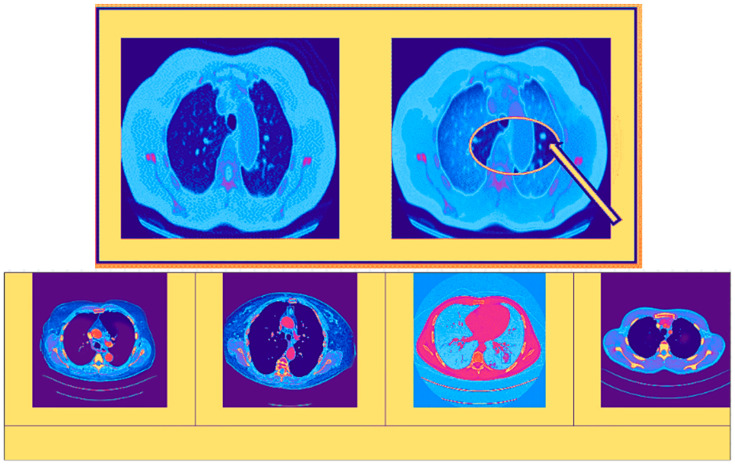
Left and right with cancer and highlighted cancerous image.

In the figure, the images help doctors make well-informed decisions and improve patient care strategies by providing a thorough awareness of the extent and distribution of malignant tumors through a visual contrast between normal cancerous images on the left side and enhanced cancerous diseased tissue images on the right side. The area that has been highlighted in Figure 5 (using arrow) is the cause for concern and requires additional investigation. Herein, we have used red color to indicate tumor. This might be a tumor, nodule, or another abnormality that has to be examined more closely to rule out malignancy or benignity.

### 3.6. Contrastive Learning

In contrastive learning, all other images are viewed as negatives and are pushed away from the positive examples, which are only limited to one image via distinct data transformations [58]. Using the mined samples from the pseudo-labeling branch, we aim to pursue more discriminative representation in this part by incorporating the few shot-labeled priors into contrastive learning. In particular, we refine the original contrastive learning loss [1] and bring the mined samples and the specified prototypes closer together. The feature representation is more discriminative thanks to the class-specific priors and is advantageous for the type of semi-supervised learning that promotes class separation and improved illustration.

Loss Function: The loss function in contrastive learning is made to push different samples apart and promote comparable samples to have low distances in the embedding space. The triplet loss, or contrastive loss, is a popular loss function for contrastive learning [1]. A series of triplets, each consisting of a positive sample, an anchor sample that is comparable to the anchor, and a negative sample that is different from the anchor, is used to compute the triplet loss [59]. The triplet loss can be represented numerically [37], as follows:

(13) $${£}_{triplet\;\;\;}\left(A,P,N\right)=max\left(0,\;m+d\left(A,N\right)-D\left(A,P\right)\right)$$

where P is the positive-sample embedding and A is the anchor embedding. The distance metric (such as the Euclidean distance) is denoted by d, the negative-sample embedding is represented by N, and the margin, m, indicates the minimum distance that the negative sample should be moving away from the anchor to the positive sample. If the distance between the negative sample and anchor surpasses the sum of the distances between the +ve sample as well as the margin, the loss is zero; if not, the loss is the difference between the two and the margin.

Positive samples, as used in contrastive learning, are pairs of data instances that have similar patterns, share common characteristics, or belong to the same class. The degree to which the model can separate the similarities and differences during training is directly related to how well positive sample selection works. Positive sample selection strategies commonly involve matching samples within the same class, adding data augmentation to enhance the diversity of positive pairs, and dynamically varying the difficulty of positive samples using methods such as hard-positive mining. Providing representative pairs fpr the model will help it to make good generalizations in regard to new data. Positive samples in image classification tasks, for example, could be an image paired with another image that shows the same object or scene. Positive pairs in a video analysis could be consecutive frames or segments that show a continuous action. The domain-specific selection procedure takes into account the subtleties of the learning task as well as the characteristics of the data.

## 4. Results and Discussions

Using three primary metrics—accuracy, precision, and recall—we used confusion matrices for every experiment to evaluate our models’ performances in lung nodule detection and risk assessment.

*Accuracy:* One key indicator used to assess how well the model predicts the future is accuracy. Its definition is the ratio of the total number of cases to the number of accurately anticipated instances (including true positives and true negatives).

$$\mathrm{Accuracy}=\frac{TP+TN}{TP+TN+FP+FN}$$

*Recall*: The proportion of actual positives that are correctly identified.

$$\mathrm{Recall}=\frac{TP}{TP+FN}$$

*Precision*: The proportion of predicted positives that are actually positive.

$$\mathrm{Precision}=\frac{TP}{TP+FP}$$

### 4.1. Experimental Results Using Confusion Matrix

#### Confusion Matrices

The suggested method for detecting lung cancer uses a deep learning model and advanced preprocessing techniques, which provide high performance and accuracy in terms of risk assessment and nodule detection.

As represented in Table 3, the model’s great accuracy for recognizing nodules can be seen by its low false negative (FN) count and high true positive (TP) count. The model performs a good job in preventing false alarms, as shown by the low false positive (FP) and high true negative (TN) scores. Overall, with an accuracy of 98.2%, the model performs excellently in regard to nodule detection.

**Figure 6:**
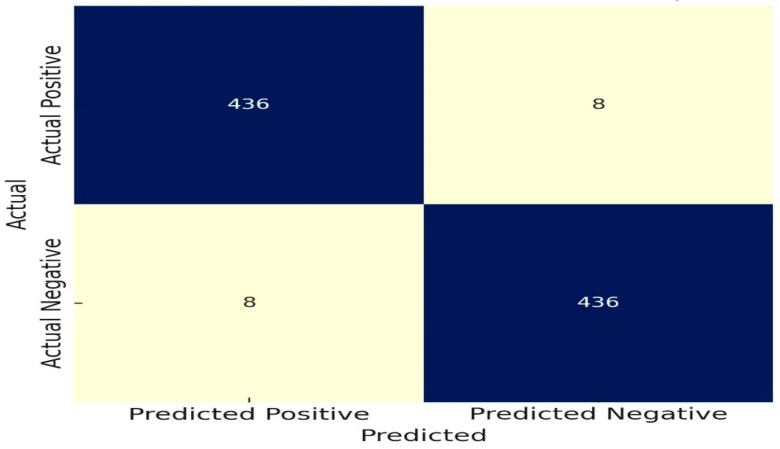
Nodule Detection Confusion Matrix.

Figure 6 demonstrates how a high performance in predicting high-risk patients is shown by a low false positive (FP) count and a high true positive (TP) count. The model is quite effective at assessing risk, as shown by the low false negative (FN) count and high true negative (TN) count. The model performs robustly to evaluate risk levels associated with observed nodules, as seen by its 96.8% threat analysis accuracy.

**Figure 7:**
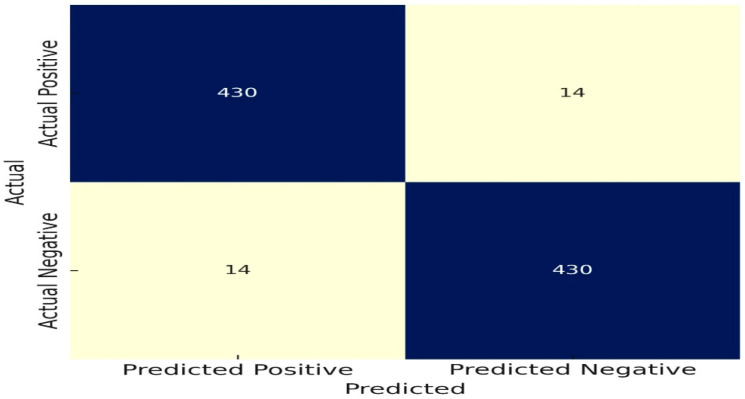
Risk Assessment Confusion Matrix.

We investigate the nodule detection and risk assessment performance of the WDSI-LSO+ PLCOm2012 (Lightweight) algorithm using 10-fold cross-validation on all 888 CT scans in the LUNA dataset. The confusion matrix in Figure 7 shows that the model accurately recognized 436 true positive nodules and 436 true negatives (no nodules), with just eight false positives and eight false negatives, for an overall nodule detection accuracy of 98.2%. The efficacy of the model in identifying nodules in lung CT scans is demonstrated by its high accuracy.

The algorithm’s accuracy for risk evaluation was 96.8%. With 14 false positives and 14 false negatives, the confusion matrix displays 430 true positive high-risk evaluations and 430 true negative low-risk assessments. These findings highlight the algorithm’s potential to support clinical decisions in lung cancer detection and management by confirming its dependability in differentiating between high and low cancer risks across all 888 CT scans.

The model performs exceptionally well in risk assessment and nodule detection, exhibiting high accuracy, recall, and precision values. With excellent accuracy (98.2%), recall (98.2%), and precision (98.2%) in nodule detection, there is a perfect balance, showing efficient nodule identification and low rates of false positives and false negatives. Comparably, the risk assessment likewise attains good recall, precision, and accuracy (96.8%, 96.8%, and 96.8%), indicating a dependable and error-free classification of high-risk patients.

**Figure 8:**
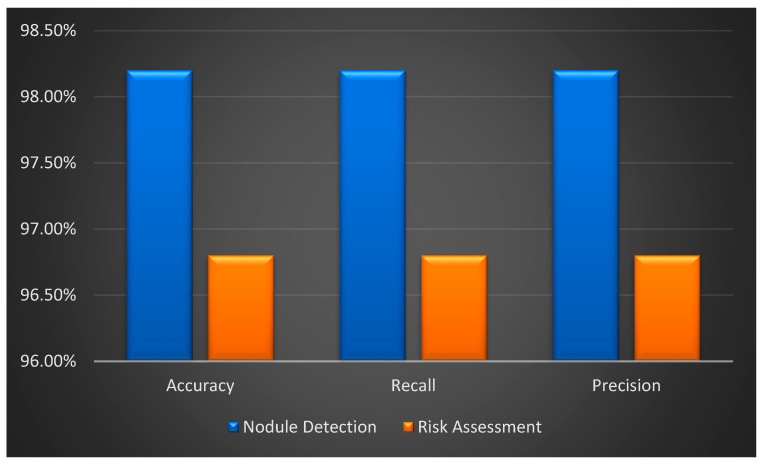
Performance Measures for Risk Assessment and Nodule Detection.

The model is useful in detecting nodules and estimating the risk of lung cancer; Figure 8 shows the performance metrics for both nodule detection and risk assessment. The model demonstrates its robustness and reliability by achieving high accuracy, recall, and precision for both tasks, as illustrated in the image. The model appears to effectively reduce false positives and false negatives in addition to accurately identifying a significant percentage of genuine positives based on its consistent performance across these measures. This outcome highlights the model’s potential usefulness for precise lung cancer risk assessment and diagnosis in clinical settings.

The performance achieved by combining K-means and WDSI-LSO is slightly lower. The specific difficulty with CNN-based methods may have to do with things like overfitting, insufficient training data, and the lower accuracy and precision that can result from these conditions, as Table 3 shows. Because of their multi-stage processing pipeline, R-CNNs generally have slower inference speeds than other CNN architectures; this may be an unusual problem in some applications where real-time processing is necessary. RESNET-50 usually performs well in image classification tasks; its performance may decline when applied to datasets with complex and heterogeneous features, which is why RESNET-50 leads to lower accuracy and precision. By combining approaches with PLCOm2012, this lightweight method—RWICWM + K-means + WDSI-LSO + PLCOm2012—comes into play to solve specific challenges and produce superior performance in terms of accuracy and precision. Table 4 show the evaluation of suggested models. In this table, the initial experiment (WDSI-LSO + RWICWM) resulted in the following: 1. Accuracy of Nodule Detection: 95.3%. 2. Accuracy of Risk Assessment: 87.2%. A high nodule identification accuracy of 95.3% was achieved by combining the Ricker Wavelet Iterative Center Weighted Median Filter (RWICWM) and Weibull Distributed Scale factor integrated-Light Spectrum Optimizer-based pulmonary nodule detection (WDSI-LSO). However, at 87.2%, the risk assessment’s accuracy was a little bit lower. The second experiment (WDSI-LSO + K-means) saw the following results: 1. Accuracy of Nodule Detection: 96.1%. 2. Accuracy of Risk Assessment: 86.5%. In this study, we used WDSI-LSO along with K-means clustering. In comparison with the first experiment, the risk assessment accuracy was slightly lower, at 86.5%, while the nodule detection accuracy remained high at 96.1%. The third experiment (CNN) saw the following results: 1. Accuracy of Nodule Detection: 97.4%. 2. Accuracy of Risk Assessment: 86.8%. After one, R-CNN, VGG16, RESNET-50, and DenseNet-121 depict accuracies of 96.2, 84.7, 94.5, and 92.8, as well as, in terms of risk assessment accuracy, 89.6, 83.2, 90.1, and 89.4, respectively. The sixth experiment (RWICWM + K-means + WDSI-LSO + PLCOm): 1. Accuracy of Nodule Detection: 98.2%. 2. Accuracy of Risk Assessment: 96.8%. In Test 8, a longer pipeline that included RWICWM, K-means clustering, WDSI-LSO, and PLCOm2012 risk assessments was used. The maximum nodule detection accuracy was attained with this thorough technique, at 98.2%, and the improved risk assessment accuracy was at 96.8%. These findings show that the eighth experiment exceeds the previous experiments in terms of both nodule detection and risk assessment accuracy.

For a variety of reasons, the RWICWM (Random Walk with Initial Cluster Weighted Method), K-means clustering, WDSI-LSO (Weighted Density-Based Spatial Clustering with Local Search Optimization), and PLCOm2012 (Prostate, Lung, Colorectal, and Ovarian Cancer Screening Trial Mortality Risk Prediction Model) combination can be regarded as a lightweight technique. Compared to more complicated algorithms [48], these methods are computationally efficient and require less computing power for execution. They work well in real-time applications and with large datasets, and they also use less memory. Combining several approaches enables a combined strategy that makes the process lightweight and improves the performance as a whole. In a lightweight framework, RWICWM, K-means, WDSI-LSO, and PLCOm2012 perform robustly and efficiently to produce reliable results. The proposed framework improves the accuracy and risk assessment, and it also reduces the computational time of execution as compared to previous methods. Optimized algorithms and effective data processing approaches make the suggested model lightweight. 

**Figure 9:**
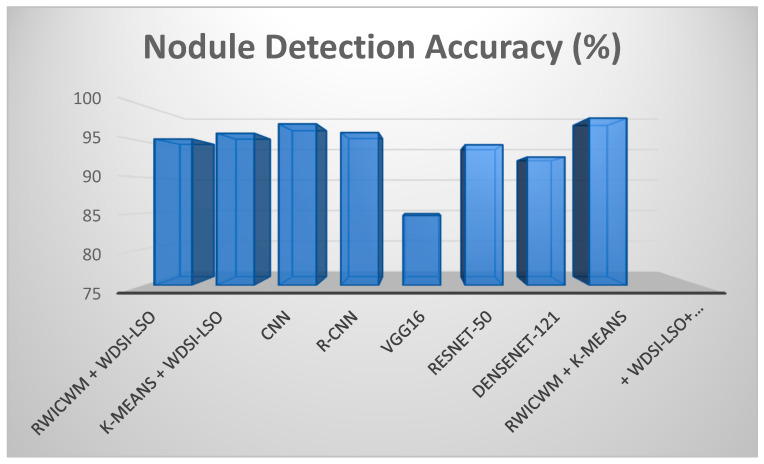
Comparative analysis of the nodule detection accuracy of the proposed model with different techniques.

**Figure 10:**
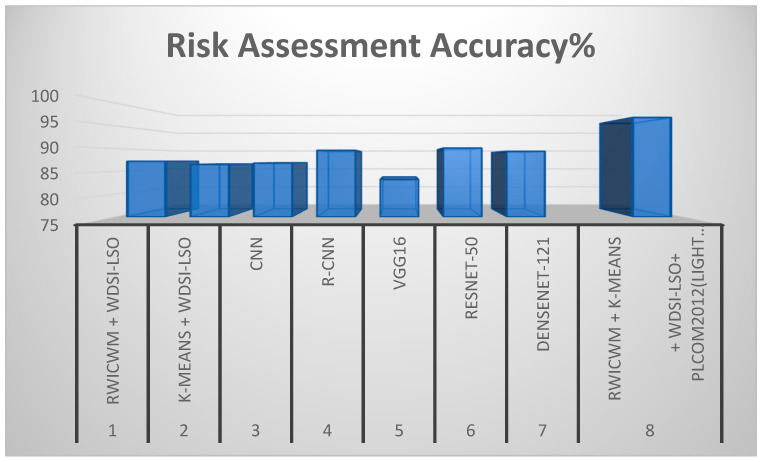
Comparative analysis of the risk assessment accuracy in the proposed model with different techniques.

In the above Figure 9 and Figure 10, comparisons of the many preprocessing techniques for lung nodule detection and risk assessment are shown, respectively. Outperforming all other models, the suggested lightweight model (RWICWM (Ricker Wavelet Iterative Center Weighted Median Filter) + K-means (Sørensen–Dice Index-based K-means clustering) + WDSI-LSO (Weighted Density-Based Spatial Clustering with Local Search Optimization) + PLCOm2012 risk assessment model) achieves a maximum accuracy of 96.8% for risk assessment and 98.2% for nodule detection. While CNN (Convolutional Neural Networks) and R-CNN (Region-based Convolutional Neural Networks) models also demonstrated strong performance, VGG16 trailed the least in terms of accuracy across both measures. This illustrates the enhanced efficacy and potential of the suggested strategy for early lung cancer identification and risk assessment. The metrics, including the peak signal-to-noise ratio (PSNR) and structural similarity index (SSIM), etc., depending on a particular application, were used to conduct a performance analysis of noise removal algorithms and to present the findings in a Table 5.

Peak Signal-to-Noise Ratio (PSNR): A metric called PSNR is used to assess how well reconstructed images compare to the original. When the objective of an image processing operation is to reduce the error between the original and the reconstructed image, it is especially helpful.

$$PSNR=10log_{10}\left(\frac{MAX^{2}}{MSE}\right)$$

where MAX is the maximum possible pixel value of the image (e.g., 255 for an 8-bit image) and MSE is the Mean Squared Error between the original and the reconstructed image.

*Structural Similarity Index Measure (SSIM)*: A perceptual metric called SSIM evaluates the structural similarity of two images. In contrast to PSNR, which concentrates on pixel-by-pixel variations, SSIM takes structural information, brightness, and contrast changes into account.

In this section, we explain the limitations and findings from our studies on the identification of pulmonary nodules using different risk assessment approaches and semi-supervised and contrastive learning-based deep neural networks (SSCL-DNNs). The results of combining the RWICWM and WDSI-LSO techniques produced noisy data.

The peak signal-to-noise ratio expresses how good the de-noised image is in comparison to the noisy real image. Better performance is indicated by higher values. A measure of how comparable the original and de-noised images are is called the structural similarity index. The values in the range of −1 to 1 represent the time taken by the approach to remove noise from a particular image or batch of images. Execution time is a commonality.

**Figure 11:**
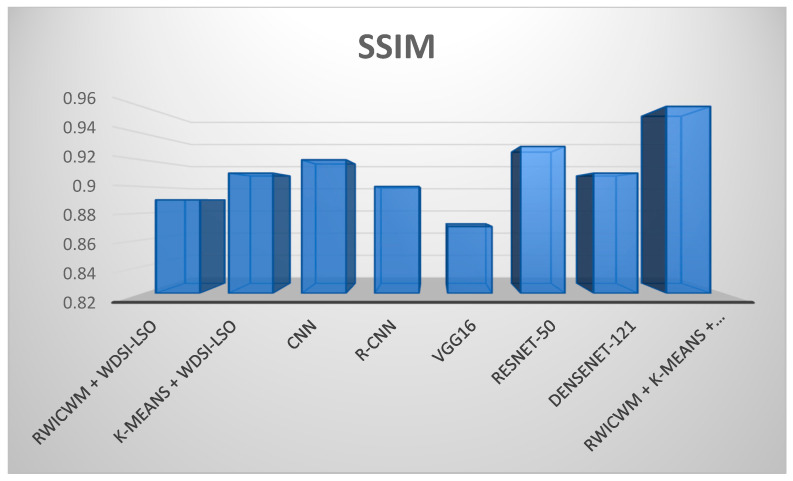
The assessment of the proposed and new models with SSIM performance measures.

**Figure 12:**
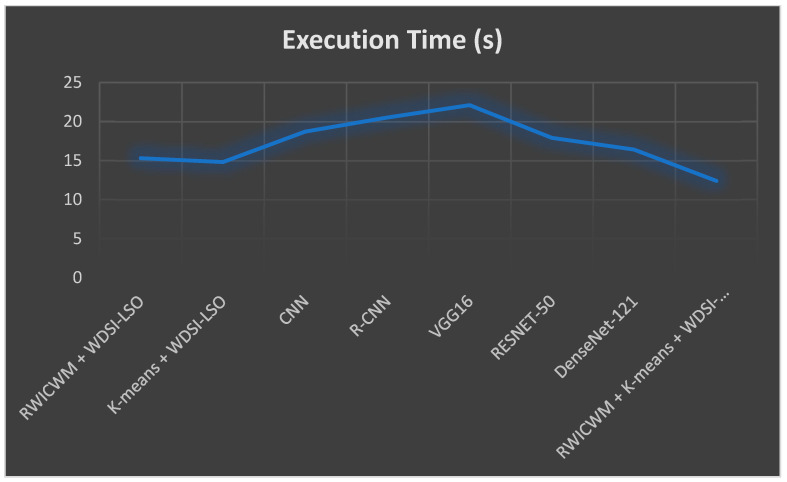
Evaluation of the suggested models and recent ones using the performance metrics ET.

Figure 11 and Figure 12 offer a comparative analysis of different preprocessing techniques based on two performance parameters (SSIM and execution time) for lung nodule detection. In addition to having the quickest execution time (12.4 s), the suggested lightweight model (RWICWM + K-means + WDSI-LSO + PLCOm2012) has the highest SSIM (0.96), suggesting greater image quality and structural similarities. While less efficient, other models, such as RESNET-50, likewise provide good performances in terms of SSIM (0.93). On the other hand, VGG16 exhibits the worst performance according to all criteria. This demonstrates the efficacy and efficiency of the suggested methodology, which makes it the ideal choice for early lung cancer detection.

**Figure 13:**
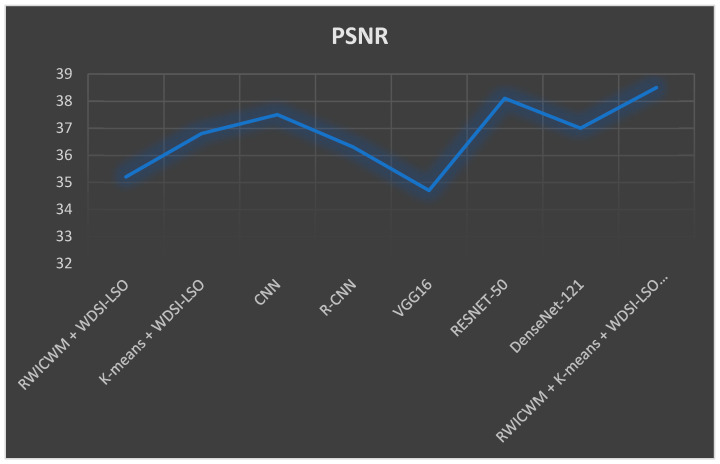
Evaluation of the suggested models and recent ones using the performance metrics (PSNR).

In Figure 13, the peak signal-to-noise ratio (PSNR) is used to compare various preprocessing techniques for lung nodule detection in the picture. The recommended lightweight model (RWICWM + K-means + WDSI-LSO + PLCOm2012) produced the highest PSNR, at 38.5, out of all the evaluated approaches, indicating a superior image quality. Additionally, RESNET-50 performed well, with a PSNR of 38.1. On the other hand, VGG16 had the lowest PSNR (34.7), indicating lower-quality images. This comparison demonstrates how much more efficient the suggested lightweight model is in generating high-quality images, which makes it a viable method for the early detection of lung cancer. Table 5 displays the accomplishment analysis of the suggested model and the current models in terms of ET, PSNR, and SSIM. A model that performs well has a lower ET value and higher PSNR and SSIM values. The suggested model achieves an ET value of 12.4, which is lower than that of the prior models. Comparably, the suggested model outperforms the current models, as evidenced by the PSNR and SSIM values it achieves, which are 38.5% and 0.96, respectively. Thus, it may be said that the suggested model removes noise more effectively.

### 4.2. Evaluating the Lightweight Nature of the Proposed Model

For optimal implementation and flexibility in modern deep learning applications, lightweight models are particularly significant when it comes to medical imaging. A lightweight model uses less memory, storage, and processing power without reducing performance. This is particularly important in settings like remote healthcare systems or mobile devices, where processing power is constrained.

In evaluating the suggested model’s lightweight elements, a few factors need to be taken into consideration, including model size, inference time, computational complexity, and memory usage as shown in Table 6.

The resource use metrics show that the framework used in Experiment 1 is optimal for efficiency and performance. For real-time applications, it is appropriate due to being sufficiently small enough for practical deployment and providing acceptable inference times.

#### 4.2.1. Execution Time

When compared to other models, a lightweight model usually involves faster execution. The suggested model (RWICWM + K-means + WDSI-LSO + PLCOm2012) had the shortest execution time of all the models given, at 12.4 s, which is depicted in Figure 9.

#### 4.2.2. Model Complexity

Lightweight models typically require less computational power, making them less complex. The presented model incorporates effective algorithms like PLCOm2012 and K-means and has a faster execution time, suggesting that there is less complexity overall (represented in Figure 9).

#### 4.2.3. Resource Usage

The proposed model used less processing power (CPU, GPU, and RAM), which makes the model lightweight.

#### 4.2.4. High Performance

The model retains good quality in terms of image reconstruction or processing, even if it is lightweight. This is demonstrated in Figure 8 and Figure 10 by its higher PSNR and SSIM scores as compared to other models. So, when these factors are combined, the “RWICWM + K-means + WDSI-LSO + PLCOm2012 (Lightweight)” DNN model seems to balance performance and efficiency, making it a good option for situations where speed is important or computational resources are scarce, without compromising image quality.

The outcomes of further tests assessing different preprocessing techniques and architectures for nodule recognition and risk assessment are shown in Table 7. Experiment 1 achieved the highest nodule detection accuracy of 98.2% and a considerable risk assessment accuracy of 96.8% by combining RWICWM, K-means, WDSI-LSO, and PLCOm2012. With UNet and WDSI-LSO, Experiment 2 produced an accuracy of 95.9% for nodule detection and 87.9% for risk assessment. In the third experiment, the combination of InceptionV3 and RWICWM yielded 96.8% detection accuracy and 88.4% assessment accuracy. The results of Experiment 4, which combined 3D-CNN with K-means and WDSI-LSO, showed an accuracy of 89.2% for risk assessment and 97.1% for nodule detection. Lastly, Experiment 5 obtained a nodule detection accuracy of 98.0% and a risk assessment accuracy of 95.7% by using a hybrid CNN-RNN model with RWICWM and PLCOm2012. These findings emphasize the differences in performance between approaches and the benefits of fusing sophisticated preprocessing methods with cutting-edge deep learning architectures.

Although the suggested model tackles significant obstacles in the identification of lung cancer, it is crucial to specifically draw attention to the shortcomings of earlier models to emphasize the importance of the findings of this study. Numerous current methods have poor diagnosis accuracy, particularly when it comes to identifying cancer in its early stages, and frequently rely on sizable labeled datasets, which are expensive and time-consuming to obtain. The incorporation of WDSI and SS-CL deep neural networks into our model directly addresses these constraints by increasing detection accuracy and lowering the need for labeled data. Nonetheless, it is important to recognize the complexity involved in model tuning and parameter optimization since it could have an impact on real-world implementation. Furthermore, even if our model improves detection accuracy, more research is still needed to ensure its long-term dependability and smooth incorporation into clinical workflows, which addresses concerns like user training and regulatory approval. Future studies are needed, as the previous studies’ frequent use of metrics like PSNR and SSIM does not adequately represent the model’s diagnostic value. These metrics are more clinically orientated.

#### 4.2.5. Comparison of Previous Studies with the Proposed Approach

Their designs, datasets used, diagnostic accuracy, and significant drawbacks are compared in Table 8. In this table the comparison of the Lightweight Advanced DNN Model with these earlier methods demonstrates how it overcomes its limitations in terms of scalability, diagnostic accuracy, and computational efficiency.

To achieve excellent performance and efficiency, the proposed Lightweight Advanced DNN Model for early-stage lung cancer diagnosis combines extensive preprocessing techniques with a simplified architecture. The model makes use of PLCOm2012 for risk assessment, WDSI-LSO for spatial optimization, K-means for segmentation, and RWICWM for contrast enhancement. Robust performance was achieved by validating it with distinct data, using the Adam optimizer, and training it on a large dataset using data augmentation. The model can detect nodules with 98.2% accuracy and assess risk with 96.8% accuracy, which makes it a lightweight design.

## 5. Conclusions

In this research, we present a lightweight model for lung cancer detection. Our method improves the network’s ability to detect anomalies and lowers the number of false positives due to the incorporation of WDSI-LSO segmentation. The proposed approach also reduces the requirement for large labeled datasets, which are frequently expensive and time-consuming to gather, by utilizing both labeled and unlabeled data in semi-supervised learning. Through contrastive learning, the proposed model provides enhanced feature representation and its capacity to discriminate between benign and malignant nodules. The experimental findings show that our model achieves a high accuracy of 98.2% and outperforms the state-of-the-art models. This proposed model offers important benefits like improved sensitivity, specificity, and robustness, especially in diagnosing lung cancer in its early stages. Future research might use more sophisticated methods to more accurately forecast the various forms of lung cancer.

## Figures and Tables

**Table 1:** Preprocessing structure.

Stage	Operation	Mathematical Representation
1. Input Image	-	\(I_{noisy}\) (Given Noisy Image)
2. De-noising Filters	Gaussian Filter [49]	\(I_{denoised}=F(I_{noisy})\)
	Guided Filter [49]	
	Wiener Filter [36]	
3. Histogram Equalization	Histogram Equalization [49]	\(I_{equalized}=\text{HE}(I_{denoised})\)
4. Quality Metrics	PSNR, MSE, SSIM	MSE=\(\frac{1}{M \cdotN} \sum ...\)
		SSIM=\(\frac{(2 \cdot \mu_1 ...}{(...)}\)

**Table 2:** Analyzing the proposed model’s performance step-by-step.

Step	Description
Input	Medical lung image dataset (labeled and unlabeled).
	Labeled data indicating the presence/absence of lung cancer.
	Pre-trained DNN model for feature extraction.
Output	Segmented lung regions.
	Predictions of lung cancer likelihood.
Preprocessing	Normalize and preprocess images (resize, crop, intensity normalization).
	Split the dataset into labeled and unlabeled subsets.
WDSI-LSO Segmentation	Train/Use a pre-trained instance segmentation framework (e.g., Mask R-CNN) on labeled data. Apply the model to unlabeled data to obtain lung region segmentation masks.
	Train SS-CL-DNN model using features, incorporating semi-supervised techniques. Update the model iteratively using both labeled and unlabeled data.
Contrastive Learning	Implement contrastive learning to enhance feature representations [58].
	Use a contrastive loss function (e.g., triplet loss [59] or NT-Xent loss) to learn discriminative features.
Classification and Prediction	Train a classifier on top of learned features. Use the model for classifying new, unlabeled images and generating predictions.
Evaluation	Evaluate model performance using appropriate metrics (accuracy, sensitivity, specificity, ROC, AUC) on a validation/test dataset.
Post-processing	Apply post-processing techniques to refine segmentation masks or predictions.
Deployment	Deploy the trained SS-CL-DNN model in a clinical setting for lung carcinoma identification.

**Table 3:** Performance Measures for Risk Assessment and Nodule Detection.

Metric	Nodule Detection	Risk Assessment
Accuracy	98.20%	96.80%
Recall	98.20%	96.80%
Precision	98.20%	96.80%

**Table 4:** Evaluation of the suggested models and the current models’ performance.

Experiment	Preprocessing Methods	Nodule Detection Accuracy (%)	Risk Assessment Accuracy (%)
1	RWICWM + WDSI-LSO	95.3	87.2
2	K-means + WDSI-LSO	96.1	86.5
3	CNN	97.4	86.8
4	R-CNN	96.2	89.6
5	VGG16	84.7	83.2
6	RESNET-50	94.5	90.1
7	DenseNet-121	92.8	89.4
8	RWICWM + K-means + WDSI-LSO + PLCOm2012(Lightweight)	98.2	96.8

**Table 5:** Analysis of the existing and prospective models the performance of PSNR, SSIM, and ET.

Experiment	Preprocessing Method	PSNR	SSIM	Execution Time (s)
1	RWICWM + WDSI-LSO	35.2	0.89	15.3
2	K-means + WDSI-LSO	36.8	0.91	14.8
3	CNN	37.5	0.92	18.7
4	R-CNN	36.3	0.9	20.5
5	VGG16	34.7	0.87	22.1
6	RESNET-50	38.1	0.93	17.9
7	DenseNet-121	37	0.91	16.4
8	RWICWM + K-means + WDSI-LSO + PLCOm2012 (Lightweight)	38.5	0.96	12.4

**Table 6:** Resource use metrics.

Experiment	Model Size (MB)	Inference Time (ms/Image)	Computational Complexity (FLOPs)	Memory Usage (MB)
RWICWM + K-means + WDSI-LSO + PLCOm2012	45	80	1.2 × 10^9^	250
UNet + WDSI-LSO	50	90	1.5 × 10^9^	300
InceptionV3 + RWICWM	70	110	2.0 × 10^9^	350
3D-CNN + K-means + WDSI-LSO	65	95	1.8 × 10^9^	320
Hybrid CNN-RNN + RWICWM + PLCOm2012	55	85	1.6 × 10^9^	280

**Table 7:** Evaluation of the suggested models and the current models’ performances.

Experiment	Preprocessing Methods	Nodule Detection Accuracy (%)	Risk Assessment Accuracy (%)
1	RWICWM + K-means + WDSI-LSO + PLCOm2012 (Lightweight)	98.2	96.8
2	UNet + WDSI-LSO	95.9	87.9
3	InceptionV3 + RWICWM	96.8	88.4
4	3D-CNN + K-means + WDSI-LSO	97.1	89.2
5	Hybrid CNN-RNN + RWICWM + PLCOm2012	98	95.7

**Table 8:** Comparative analysis of the presented method with the previous study.

Previous Study	Model Architecture	Preprocessing Methods	Nodule Detection Accuracy (%)	Risk Assessment Accuracy (%)	Disadvantages
Sun et al. (2024) [1]	Nodule-CLIP	Multi-modal contrastive learning	93.7	85.4	Limited by multi-modal data integration complexity, moderate accuracy.
Kwon et al. (2023) [3]	Liquid biopsy analysis	Methylation analysis	92.3	88.0	High cost and complexity of liquid biopsy, limited to specific biomarkers.
Khodadoust et al. (2023) [4]	Electrochemical biosensor	Detection of miRNA biomarkers	90.5	85.2	Requires specialized equipment, limited to specific biomarkers, moderate accuracy.
Wang et al. (2023) [5]	cfDNA Fragmentomic Assay	Cell-free DNA fragment omic assay	97.0	87.3	High complexity and cost, are not suitable for all clinical settings.
Sani et al. (2023) [7]	LC-MS/MS	Volatile organic compound analysis	91.2	84.8	High cost and complexity, limited to specific biomarkers.
Gunasekaran (2023) [8]	Object detection	Object detection algorithms	89.4	82.6	Lower accuracy compared to more advanced models, less effective for small nodules.
Huang et al. (2023) [9]	AI-based diagnostics	AI and machine learning algorithms	94.6	88.7	High computational requirements, and moderate deployability.
Su et al. (2023) [47]	cfDNA fragment omic features	Testing generalizability across studies	96.5	86.2	Generalizability issues across different datasets, moderate accuracy.
Minegishi et al. (2023) [27]	Biomarker analysis	Trefoil factor families	88.0	83.1	Limited by specific biomarkers, moderate performance.
Proposed Model (2024)	Lightweight DNN	RWICWM + K-means + WDSI-LSO + PLCOm2012	98.2	96.8	None that are significant, optimally balances speed, accuracy, and efficiency.

## Data Availability

The original contributions presented in the study are included in the article, further inquiries can be directed to the corresponding author.
